# Hepatic perivascular epithelioid cell tumor resembling hepatic adenoma and hepatocellular carcinoma on preoperative imaging: a case report

**DOI:** 10.3389/fonc.2024.1292313

**Published:** 2024-02-01

**Authors:** Dongling Zhu, Shuang Song, Dongdong Wang, Dong Kuang, Siyuan Cheng, Jianyuan Zhou, Sijuan Zou

**Affiliations:** ^1^ Department of Nuclear Medicine and PET, Tongji Hospital, Tongji Medical College, Huazhong University of Science and Technology, Wuhan, China; ^2^ Department of Pathology, Tongji Hospital, Tongji Medical College, Huazhong University of Science and Technology, Wuhan, China

**Keywords:** PEComa, melanocytic, smooth muscle, ^18^F-FDG, ^68^Ga-FAPI

## Abstract

Perivascular epithelioid cell tumor (PEComa), an uncommon mesenchymal neoplasm, arises from specialized perivascular epithelioid cells exhibiting distinct features of smooth muscle and melanocytic differentiation with unpredictable behavior. PEComa tends to occur more commonly in the uterus and kidneys; its occurrence in the liver is exceedingly rare. We presented a case of a 29-year-old woman with hepatic PEComa and evaluated the tumor with MRI, integrated ^18^F-fluorodeoxyglucose (FDG), and ^68^Ga-fibroblast activation protein inhibitor (FAPI) PET/CT scans at presentation. The patient had a history of intermittent utilization of oral contraceptive drugs for several years. An abdominal ultrasound in a physical examination from an outside institution revealed a mass in the liver. A contrast-enhanced abdominal MRI revealed restricted diffusion on diffusion-weighted imaging (DWI) and rapid contrast enhancement and washout patterns in the hepatic lesion, suggesting hepatic adenoma (HA) or hepatocellular carcinoma (HCC). Further assessment was carried out using ^18^F-FDG and ^68^Ga-FAPI PET/CT scans. The hepatic lesion was non-FDG avid, whereas increased tracer uptake was observed on the ^68^Ga-FAPI PET/CT. Subsequently, laparoscopic partial resection of liver segment V was performed. Immunohistochemical analyses demonstrated positive staining for HMB45, Melan-A, and SMA while showing negative results for AFP, glypican-3, hepatocyte, and arginase-1. The results were indicative of a hepatic PEComa diagnosis based on these findings. We also review the current literature on the clinical characteristics, pathological features, and challenges in the diagnosis of hepatic PEComa.

## 1 Introduction

Perivascular epithelioid cell tumor (PEComa) is an infrequent but distinct type of mesenchymal neoplasm. The PEComa family mainly includes the renal and extrarenal types of angiomyolipoma (AML), the pulmonary and extrapulmonary types of clear-cell “sugar” tumors (CCST) or lymphangioleiomyomatosis (LAMs), and the PEComa not otherwise specified (PEComa NOS) (1). In 1992, Bonnetti initiated the concept of perivascular epithelioid cells (PEC) (2). Zamboni subsequently coined the term PEComa in 1996 to designate the group of tumors distinguished by these specific cell types and further elucidated their pathological features (3). Typically, spindle cells may be found adjacent to epithelioid cells, and the PEComa may accumulate a great amount of lipids (4). Previous research suggested a potential association between PEComa and dysfunction of TSC complex 1 and complex 2, particularly TSC2, which negatively regulated mTORC1 and contributed to tumor development (5, 6). Immunohistochemical analysis reveals positive staining for melanocytic-related biomarkers HMB45 and Melan-A and smooth muscle marker SMA (7, 8). PEComas are tumors with unpredictable behavior. Primary hepatic PEComa is extremely rare. Data regarding hepatic PEComa evaluation with PET/CT are limited. This case highlights the diagnostic challenges regarding identifying and thoroughly evaluating hepatic PEComa preoperatively. As indicated by our case, ^18^F-FDG and ^68^Ga-FAPI dual-tracer PET/CT imaging may play a significant role in the detection, differentiation, and whole-body evaluation of hepatic PEComa.

## 2 Case presentation

**Figure 1:**
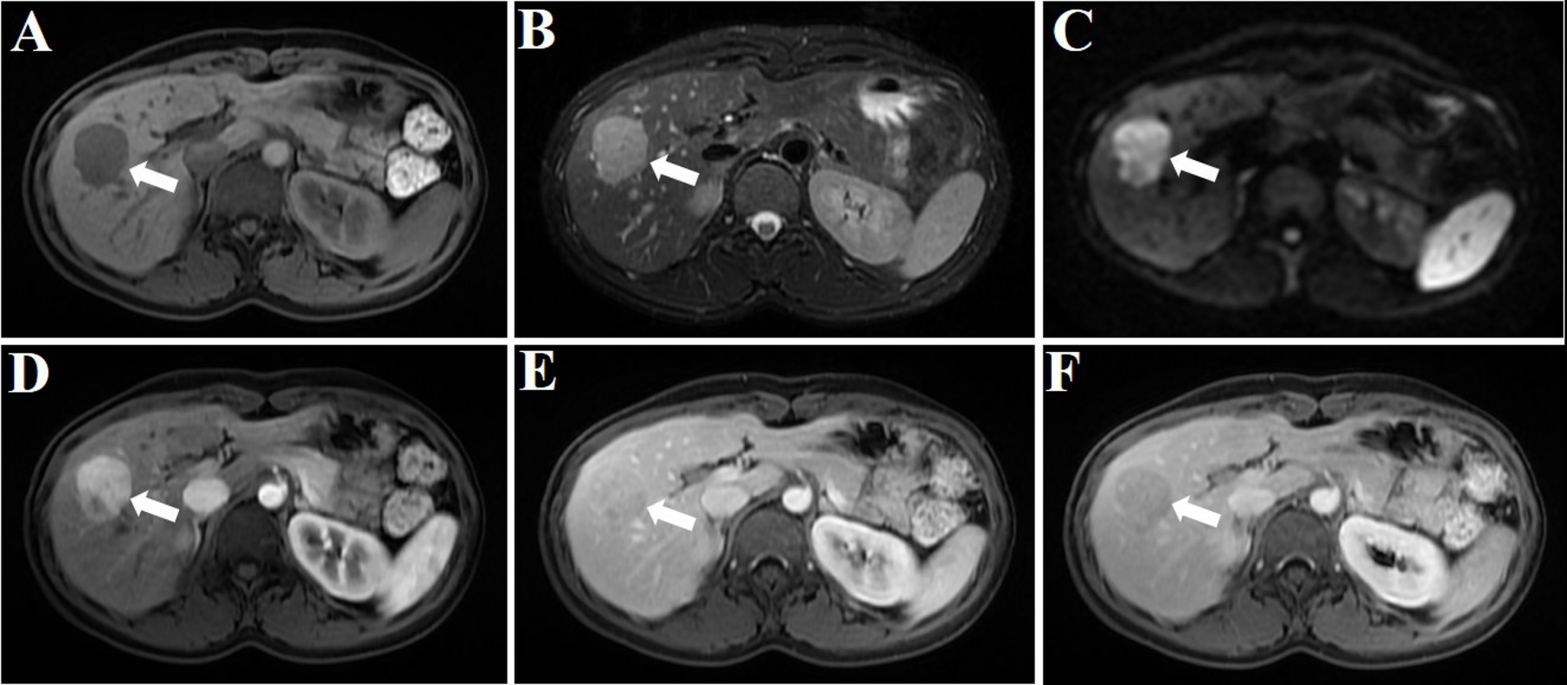
MRI plain scan, DWI, and enhanced image of the hepatic PEComa are presented. The hepatic PEComa exhibited a slightly prolonged T1 signal **(A)** and a slightly prolonged T2 signal **(B)**. The DWI showed restricted diffusion **(C)**. The enhancement pattern was characterized by fast-in and fast-out. During the arterial phase, a significant enhancement was observed, with a considerably higher degree of enhancement compared to the adjacent normal liver parenchyma **(D)**. Subsequently, in the portal vein phase **(E)** and delayed phase **(F)**, the enhancement gradually subsided and ultimately became less pronounced than the surrounding liver parenchyma (white arrows indicate the tumor).

During a routine medical examination, an abdominal ultrasound was performed on a 29-year-old female patient with no tumor history. Incidentally, a focal solid hypoechoic lesion measuring 3.5 cm in size was discovered in the right lobe of the liver. A review of the medication use history revealed that she had been taking oral contraceptives for several years. Her laboratory data indicated normal values of coagulation and liver function. The tumor marker tests, including α-fetoprotein, carcinoembryonic antigen, Des-γ-carboxyprothrombin, and carbohydrate antigen 19-9, showed values within the normal range. MRI demonstrated a 3.5-cm-sized circular lesion displaying slightly prolonged T1 and T2 signals in the liver with high signal intensity on diffusion-weighted imaging (DWI). Moreover, arterial enhancement was observed with rapid clearance during the delayed phase (**Figure 1**). With these imaging characteristics and the medication use history of oral contraceptives, the lesion was highly suspicious for liver tumors of hepatic adenoma (HA) or hepatocellular carcinoma (HCC).

**Figure 2:**
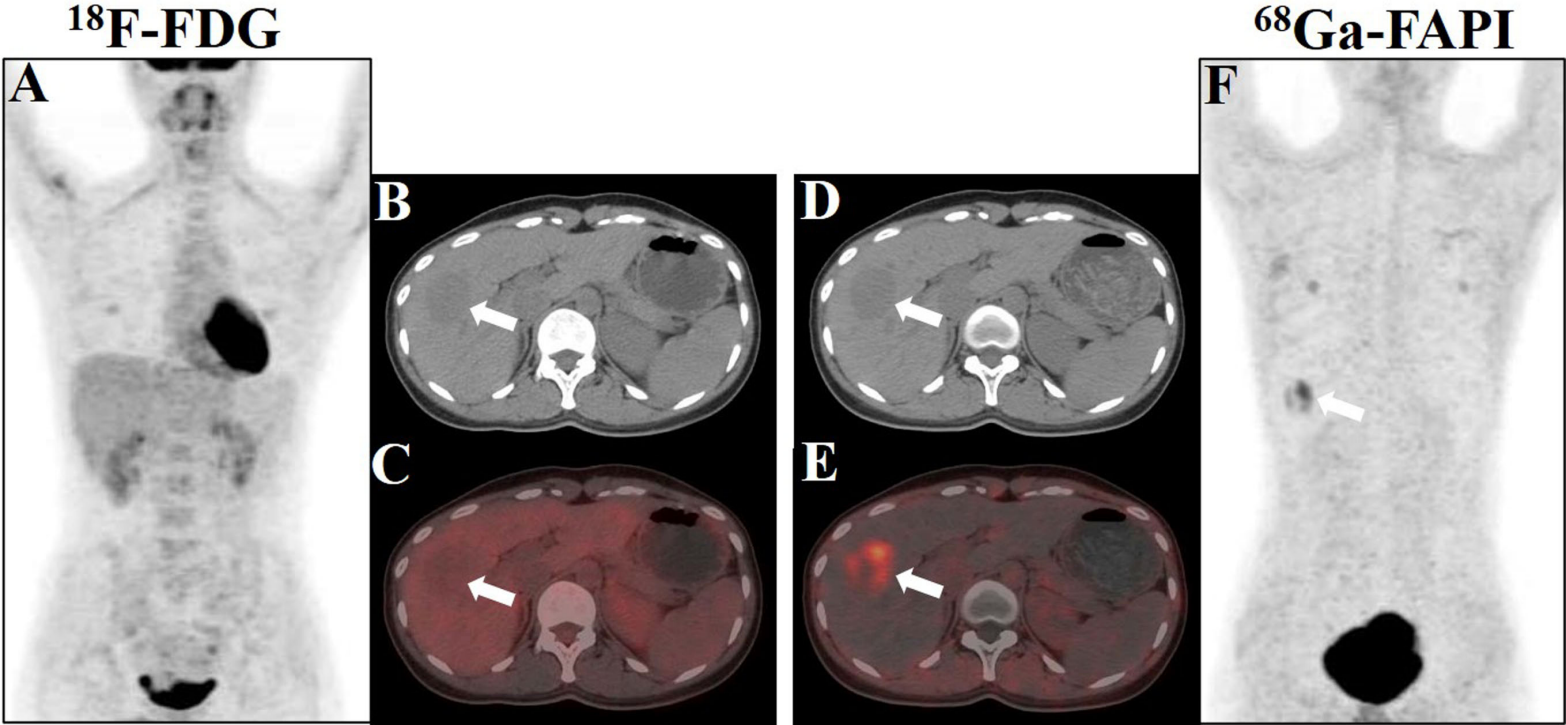
Showcase hepatic PEComa images obtained from ^18^F-FDG and ^68^Ga-FAPI PET/CT scans. **(A)** The maximum intensity projection (MIP) image generated from the FDG scan revealed a lack of tumor uptake in the liver. **(B**, **C)** Axial images acquired from CT and fused FDG PET/CT scans exhibited a mildly hypodense lesion in liver segment V, accompanied by minimal tumor radioactivity uptake, characterized by SUVmax = 1.31 and TBRmax = 0.83. **(D**, **E)** Axial images derived from CT and fused FAPI PET/CT scans demonstrated robust tumor radioactivity uptake at the same location, with SUVmax = 5.28 and TBRmax = 10.56. **(F)** The MIP image generated from the FAPI scan displayed avid tumor uptake in the liver (white arrows indicate the tumor).

An ^18^F-FDG PET/CT scan was performed for further whole-body evaluation. There was no focal activity typical of hypermetabolic malignancy on the MIP image (**Figure 2A**). The transaxial CT and FDG PET/CT images revealed a well-defined, round, slightly hypodense lesion in the lower portion of the anterior right liver lobe. The lesion was non-FDG avid with an SUVmax of 1.31 (in comparison to the normal hepatic SUVmax of 1.58), and the maximum tumor-to-background ratio (TBRmax) was 0.83 (**Figures 2B, C**). Next, the patient was enrolled in our hospital’s institutional review board-approved clinical trial utilizing ^68^Ga-fibroblast activation protein inhibitor (FAPI) PET/CT to rule out the non-FDG avidity of low-grade liver tumors. The patient provided written consent for participation. The ^68^Ga-FAPI PET/CT scan was conducted 6 days following the ^18^F-FDG PET/CT scan. Interestingly, ^68^Ga-FAPI PET/CT MIP, transaxial CT, and transaxial fused images (**Figures 2D–F**) demonstrated intense FAPI uptake (SUVmax, 5.28; TBRmax, 10.56) in the hepatic lesion. No other masses were observed, and there was no abnormally increased uptake of FDG and FAPI in the remaining organs.

**Figure 3:**
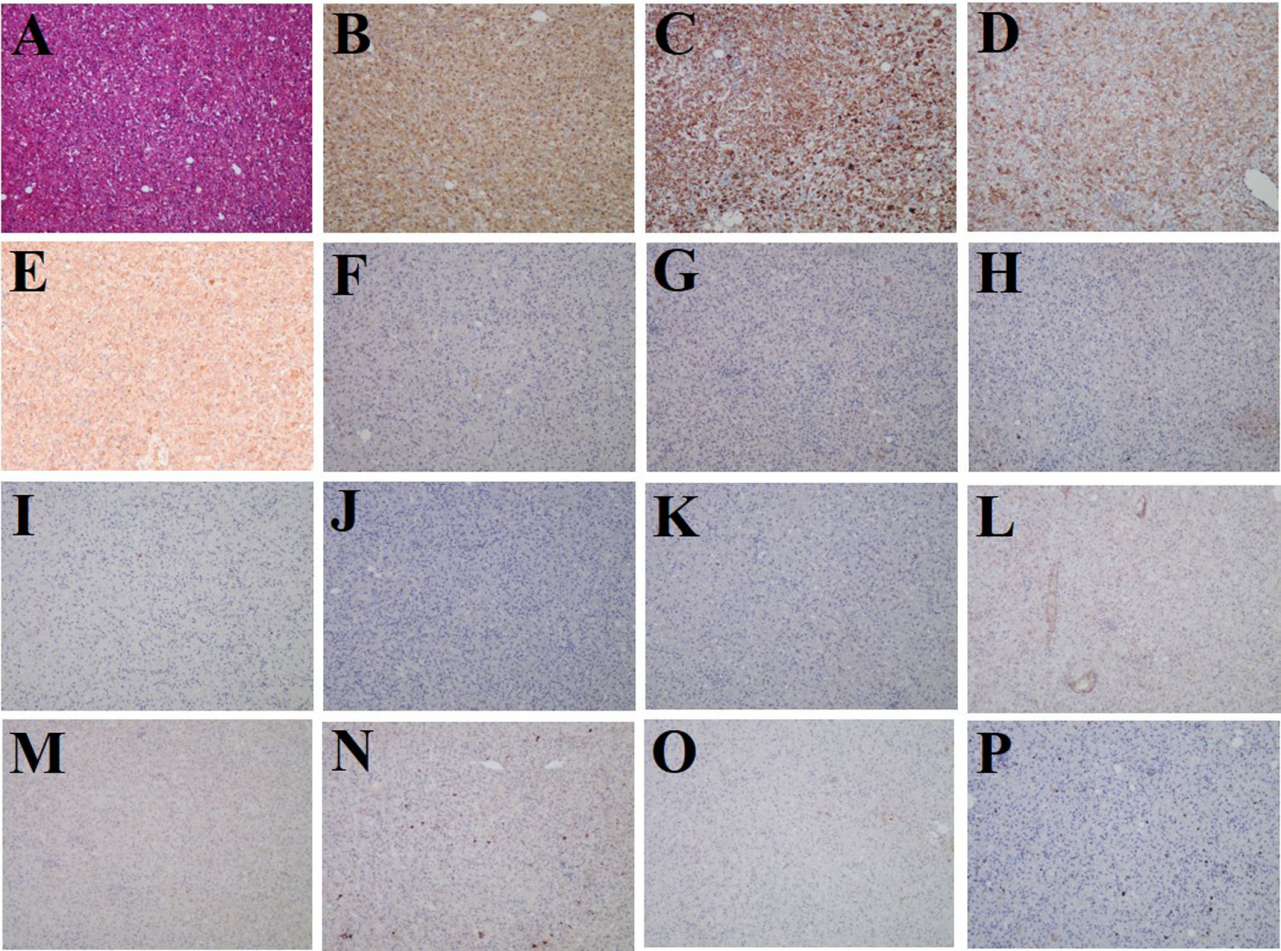
Histological and immunostaining analysis of hepatic PEComa. **(A)** Histological examination using hematoxylin and eosin (HE) staining revealed hepatic PEComa. Immunohistochemical markers HMB45 **(B)** and Melan-A **(C)** for melanocytic, SMA **(D)** for smooth muscle, and FAP **(E)** exhibited positive expression. Conversely, immunohistochemical markers glypican-3 **(F)**, hepatocyte **(G)**, arginase-1 **(H)**, and AFP **(I)** for HCC, CK19 **(J)** and CK7 **(K)** for intrahepatic cholangiocarcinoma, desmin **(L)** for muscle tissue, SOX-10 **(M)** and S-100 **(N)** for nerve and melanoma, and PCK **(O)** for broad-spectrum epithelial tumors demonstrated negative expression. **(P)** the Ki-67 LI was merely 3%, indicating a low proliferation rate and benign biological characteristics.

Subsequently, the patient underwent laparoscopic partial hepatectomy of segment V, and hematoxylin and eosin (HE) staining indicated predominantly epithelioid tumor cells without significant atypical features, tumor necrosis, or pathological mitosis (**Figure 3A**). Immunohistochemistry analyses yielded positive results for HMB45, Melan-A, SMA, and FAP (**Figures 3B–E**), but negative for glypican-3, hepatocyte, arginase-1, AFP, CK19, CK7, desmin, SOX-10, S-100, and PCK (**Figures 3F–O**). The hepatic PEComa also presented a low Ki-67 labeling index (LI) of 3% (**Figure 3P**). Thus, the final diagnosis was primary hepatic PEComa with no indications of malignancy. At 12 months of follow-up, the patient remained asymptomatic and showed no signs of disease recurrence on MRI monitoring.

## 3 Discussion

PEComa, an uncommon mesenchymal tumor deriving from perivascular epithelioid cells, demonstrates a significant gender-related disparity in the incidence rates, with women being affected nearly four times more frequently than men (9, 10). Nevertheless, it is crucial to recognize that these data might be susceptible to bias due to the limited sample size, potential geographical and population variations, and the rarity of the disease itself. Based on the distinct distribution of PEC across tissues, PEComa primarily encompasses AML, CCST, LAMs, and PEComa NOS in other tissue sites. Likewise, the diverse distribution of PEC in tissues gives rise to some variations in clinical manifestations, pathological alterations, and differential diagnosis for each type. Intra-abdominal PEComa may present with palpable masses that cause pain due to compression of adjacent tissues or nerves (11). Cutaneous PEComa may cause bleeding symptoms (12), while lung involvement can lead to coughing and respiratory difficulties (13). PEComa occurring in the liver is considered to be very rare. According to reference, more than 200 cases of hepatic PEComa have been reported, but most of them were the type of hepatic AML (14). While AML predominantly affects the kidneys, it can also manifest in extrarenal organs, including the liver. Hepatic AML primarily consists of fat, blood vessels, and PEC, and if PEC largely predominates, it can be defined as a type of PEComa called hepatic epithelioid AML (HEAML). A recent study conducted in 2023 identified an additional 113 cases of HEAML as a distinct subtype of PEComa (15). With the increasing clinical understanding of PEComa, it becomes evident that the existing cases may not precisely reflect the actual incidence of hepatic PEComa. Hepatic PEComa typically does not experience any discomfort but sometimes exhibits some nonspecific symptoms, such as dull upper right abdominal pain, abdominal bloating, nausea, vomiting, and diarrhea related to the gastrointestinal system (16, 17). As in our case, the hepatic PEComa was accidentally found at a medical checkup with no initial clinical manifestation. It is interesting to note that the patient had been using oral contraceptives for several years. Prior research established a notable association between oral contraceptives and liver tumors, particularly HA (18), which can lead to complications such as malignant transformation to HCC. Furthermore, HA primarily affects women of childbearing age who are taking oral contraception (19). However, cases of hepatic PEComa with a medication use history of oral contraceptives have been rarely reported.

The molecular mechanisms underlying PEComa remain elusive; however, the TSC1-TSC2/mTOR signaling pathway has been identified as a significant molecular pathway implicated in tumorigenesis by promoting the dysfunction of TSC1-TSC2, with particular emphasis on TSC2 mutations that activate mTOR. A comprehensive molecular analysis revealed TSC2 mutations in eight out of 13 cases of PEComa (62%). Excluding TFE3 fusion cases increased the proportion of PEComa cases with TSC2 mutations to 80% (eight out of 10 cases) (20). Another molecular mechanism driving PEComa development involves TFE3 fusion. Among studies investigating the impact of TFE3-related molecular pathways on PEComa, TFE3 fusion was detected in nine out of 38 cases (24%) of PEComa (20). SFPQ/PSF emerged as the most commonly recurring gene in PEComa cases with TFE3 rearrangement (21). Notably, no occurrence of TSC2 mutations was observed in the presence of TFE3 rearranged PEComa, highlighting a high degree of mutual exclusion between TFE3 fusion and TSC2 mutation (20, 22).

The preoperative diagnosis of PEComa by imaging is challenging. In a study by Chen et al., the radiologic findings of hepatic PEComa were analyzed in seven cases (23). Among four of the patients who underwent contrast-enhanced scans, three showed enhancement during the arterial phase, but imaging manifestations during the venous and delayed phases varied. In our case, the hepatic lesion demonstrated distinct arterial phase enhancement followed by gradual clearance in the venous and delayed phases (**Figure 1**). The presence of a rich arterial blood supply and relatively regular morphology of the liver mass should be distinguished from other liver masses, such as HCC, HA, and focal nodular hyperplasia (FNH) (15). While PEComa often contains fat, this particular hepatic PEComa is primarily composed of epithelioid cells, with no detectable fat component observed, which also indicates the subtype of HEAML. The imaging feature of fast-in and fast-out enhancement exhibited by this hepatic PEComa makes it challenging to differentiate it from HCC. In the case of most HA, the lesion may show faint enhancement in the arterial phase and lack gradual clearance, while some atypical HA cases may also display the imaging features of fast-in and fast-out enhancement. Especially when considering the history of using oral contraceptives, the case can be easily misdiagnosed as HA. FNH, on the other hand, often presents a scar in the center of the mass without enhancement in the arterial phase. The imaging features of hepatic PEComa are diverse and could be mimicked by other liver masses. Therefore, the preoperative diagnosis of hepatic PEComa poses a challenge.

PET/CT imaging might play a role in distinguishing between benign and malignant PEComa. Previous studies identified that positive ^18^F-FDG uptake was predictive of an aggressive disease. Malignant PEComa tends to display high radioactive uptake on ^18^F-FDG PET/CT; however, benign PEComa is commonly non-FDG avid or with low FDG uptake (24). In addition, the use of FDG PET/CT in liver cancer is challenging because of the degree of heterogeneity of the tumor. As a tumor stroma imaging agent, ^68^Ga-FAPI PET/CT offers enhanced imaging effectiveness when compared to ^18^F-FDG in various tumors, particularly those with low ^18^F-FDG uptake or tumors located within tissues or organs exhibiting high physiological uptake of ^18^F-FDG (25, 26). In a case of renal malignant PEComa reported by Zhang et al., ^68^Ga-FAPI demonstrated superior radioactive uptake compared to ^18^F-FDG in that girl (27). We presented the initial instance of benign hepatic PEComa revealed on ^68^Ga-FAPI PET/CT, demonstrating greater tumor-to-background contrast compared to ^18^F-FDG PET/CT in visualizing the tumor (**Figure 2**). Intense uptake of ^68^Ga-FAPI was found in various sarcomas, possibly owing to the abundant interstitial constituents in these neoplasms (28, 29). Based on the fact that PEComa is one type of mesenchymal tumor, we performed further immunohistochemical analysis to validate the heightened expression of FAP in this neoplasm (**Figure 3E**). Furthermore, in contrast to ^18^F-FDG PET, ^68^Ga-FAPI PET demonstrates remarkable image contrast accompanied by minimal background activity across the entirety of the body, which enables the detection of aggressive PEComa with multiorgan involvement. Accurate evaluation of organ involvement is crucial for predicting the prognosis and making therapeutic decisions in patients with PEComa. As indicated by our case, ^18^F-FDG and ^68^Ga-FAPI dual-tracer PET/CT imaging may play a significant role in the detection, differentiation, and whole-body evaluation of hepatic PEComa. Additionally, the high expression of FAP in PEComa lesions suggests that the use of FAP-targeted radiotherapy for aggressive PEComa is promising; this has already been shown in other types of tumors (30).

The definitive diagnosis of hepatic PEComa primarily relies on pathological examination. A fine needle aspiration biopsy is commonly employed for diagnosis; however, limited tissue availability often poses challenges in achieving accurate results or may lead to false negatives (31). In our case, liver segment resection was performed, and the excised specimen was meticulously examined under a microscope. Recent guidelines from the World Health Organization (WHO) highlight that PEComa is a unique type of mesenchymal tumor characterized by specific smooth muscle and melanocytic markers, notably SMA, Melan-A, and HMB-45 (32). Therefore, in addition to the cellular morphological features, immunohistochemistry plays a crucial role in the differential diagnosis of hepatic PEComa. In this liver mass, intense staining of HMB45, Melan-A, and SMA was observed, which was consistent with the diagnosis of hepatic PEComa (**Figures 3B–D**). Immunohistochemistry was performed to differentiate liver tumors, and glypican-3, hepatocyte, arginase-1, and AFP were used for HCC (**Figures 3F–I**), CK19 and CK7 for intrahepatic cholangiocarcinoma (**Figures 3J, K**), desmin for muscle tissue (**Figure 3L**), SOX-10 and S-100 for nerve and melanoma (**Figures 3M, N**), and PCK for broad-spectrum epithelial tumors (**Figure 3O**). All these markers showed negative staining. Additionally, the low proliferation rate of Ki-67 LI (3%) indicated benign biological characteristics (**Figure 3P**).

Currently, there is no consensus or universally accepted criteria for the differential diagnosis between benign and malignant PEComa. According to Folpe et al., malignant PEComas typically exhibit certain features, including a tumor size larger than 5 cm, elevated nuclear grade, increased cellular density, a mitotic rate surpassing 1/50 high-power fields, necrosis, infiltration into the adjacent normal parenchyma, and invasion of blood vessels (33). The presence of two or more of these characteristics usually indicates a malignant PEComa. However, none of these criteria were met in this specific case. Surgery remains the primary treatment option for PEComa. However, for patients with advanced metastasis, there is currently no established, effective medical approach. Some studies suggest that oral mTOR inhibitors may have varying degrees of effectiveness in treating advanced-stage PEComa patients, while a few cases show no significant treatment response (34–36). A retrospective study indicated that mTOR inhibitor therapy exhibits a higher objective response rate and longer disease progression-free survival in comparison to chemotherapy and VEGFR inhibitor therapy (37). Nonetheless, further research, particularly prospective cohort studies, is still required to provide effective treatment options for patients with advanced PEComa.

## 4 Conclusion

The authors present a patient with hepatic PEComa evaluated by MRI, integrated ^18^F-FDG, and ^68^Ga-FAPI PET/CT. Although the preoperative diagnosis of PEComa is challenging, the possibility of PEComa should be kept as one of the differentials in interpreting imaging studies in patients with hepatic mass. In this case report, we present the first ^68^Ga-FAPI PET/CT observations of benign hepatic PEComa, unveiling a higher tumor-to-liver ratio in contrast to ^18^F-FDG PET/CT. ^18^F-FDG and ^68^Ga-FAPI dual-tracer PET/CT imaging can have a substantial impact on the detection, differentiation, and whole-body evaluation of hepatic PEComa. The underlying mechanism of radiotracer uptake of ^68^Ga-FAPI and its prospective implications for the diagnosis and therapeutic interventions for hepatic PEComa necessitate further exploration.

## Data availability statement

The original contributions presented in the study are included in the article. Further inquiries can be directed to the corresponding authors.

## Ethics statement

The studies involving humans were approved by the Ethics Committee of Tongji Hospital, Tongji Medical College, Huazhong University of Science and Technology. The studies were conducted in accordance with the local legislation and institutional requirements. The participants provided their written informed consent to participate in this study. Written informed consent was obtained from the individual(s) for the publication of any potentially identifiable images or data included in this article.

## Author contributions

DZ: Data curation, Funding acquisition, Writing – original draft, Project administration. SS: Resources, Writing – review & editing. DW: Resources, Writing – review & editing. DK: Data curation, Writing – review & editing. SC: Data curation, Writing – review & editing. JZ: Data curation, Writing – review & editing. SZ: Supervision, Writing – review & editing.
